# Development of a One-Step SYBR Green I Real-Time RT-PCR Assay for the Detection and Quantitation of Araraquara and Rio Mamore Hantavirus

**DOI:** 10.3390/v5092272

**Published:** 2013-09-19

**Authors:** Alex Martins Machado, William Marciel de Souza, Michelly de Pádua, Aline Rafaela da Silva Rodrigues Machado, Luiz Tadeu Moraes Figueiredo

**Affiliations:** Virology Research Center, School of Medicine in Ribeirão Preto, University of São Paulo, Av. Bandeirantes n.3900, Monte Alegre, Ribeirão Preto, São Paulo14049-900, Brazil; E-Mails: wmarciel@hotmail.com (W.M.S.); michellydepadua@hotmail.com (M.P.); alinerafaelasr@yahoo.com.br (A.R.S.R.M.); ltmfigue@fmrp.usp.br (L.T.M.F.)

**Keywords:** Hantaviruses, Araraquara virus, Rio Mamore virus, real-time quantitative RT-PCR, SYBR green I

## Abstract

Hantaviruses are members of the family *Bunyaviridae* and are an emerging cause of disease worldwide with high lethality in the Americas. In Brazil, the diagnosis for hantaviruses is based on immunologic techniques associated with conventional RT-PCR. A novel one-step SYBR Green real-time RT-PCR was developed for the detection and quantitation of Araraquara hantavirus (ARAV) and Rio Mamore hantavirus (RIOMV). The detection limit of assay was 10copies/µL of RNA *in vitro* transcribed of segment S. The specificity of assay was evaluated by melting curve analysis, which showed that the Araraquara virus amplified product generated a melt peak at 80.83 ± 0.89 °C without generating primer-dimers or non-specific products. The assay was more sensitive than conventional RT-PCR and we detected two samples undetected by conventional RT-PCR. The one-step SYBR Green real-time quantitative RT-PCR is specific, sensible and reproducible, which makes it a powerful tool in both diagnostic applications and general research of ARAV and RIOMV and possibly other Brazilian hantaviruses.

## 1. Introduction

Hantaviruses are members of the *Bunyaviridae* family and possess a trisegmented negative-strand RNA genome, categorized according to small (S), medium (M) and large (L) segments [1]. Hantaviruses are transmitted to humans by the inhalation of aerosols of excreta or direct contact with infected rodents, although human-to-human transmission has been documented only for the Andes virus in South America [2]. Severe diseases caused by hantaviruses are a serious public health problem worldwide. In the Americas, hantavirus infections produce a severe disease known as Hantavirus Pulmonary Syndrome (HPS), which is characterized by fever, respiratory failure, shock and a high case fatality ratio [1,2]. 

The first HPS cases in Brazil were diagnosed in 1993. Since then, approximately 1,600 HPS cases have been reported, with a 39% case fatality ratio [3]. Currently six hantaviruses are found in several species of wild rodents in Brazil: Juquitiba virus (*Oligoryzomys* *nigripes*); Araraquara virus (ARAV) (*Necromys lasiurus*); Castelo dos Sonhos virus (*Oligoryzomys* aff. *moojeni* and *Oligoryzomys utiaritensis*); Laguna Negra-like virus (*Calomys* aff*. callosus* and *Calomys laucha*); Anajatuba virus (*Oligoryzomys fornesi*) and Rio Mamore virus (RIOMV) (*Oligoryzomys* sp) [1,4,5,6].

Serological methods are commonly used for hantavirus diagnosis, including enzyme-linked immunosorbent assays (ELISAs), immunofluorescence assays and immunoblot assays [1,7,8]. Additionally, hantavirus isolation in Vero E6 cell cultures and detection of hantavirus antibodies by plaque reduction neutralization are also used for diagnosis. Nonetheless, both methods require labor-intensive, time-consuming and biosafety level 3 conditions, although these are hardly used in diagnostics [1,7].

Nucleic acid-based methods, such as polymerase chain reaction preceded by reverse transcription (RT-PCR), are suitable for the detection of hantaviruses [9,10,11]. In Brazil, a conventional RT-PCR was able to detect Araraquara hantavirus genomes in blood and tissue samples of humans and rodents [12]. Nevertheless, molecular techniques can evolve into faster, more sensitive methods that allow for the detection and quantitation of hantaviruses in several samples. 

Recently, real-time RT-PCR assays have been developed using TaqMan probes for the diagnosis and/or quantification of hantaviruses such as Puumala virus, Andes virus, Hantaan virus, New York virus, Tula virus, Seoul virus, Dobrava virus and Sin Nombre virus [10,11,13,14,15]. In addition, a real-time RT-PCR assays using SYBR Green system for diagnosis of Dobrava virus in patients with HFRS were developed [11]. In Brazil, using this same method, a real-time RT-PCR for detection of Juquitiba virus infection in rodents captured in the Atlantic rainforest was developed, but did not allow for viral quantitation [12]. 

In summary, real-time RT-PCR has many advantages over conventional RT-PCR, including rapidity, higher sensitivity and quantitative measurement [9]. Therefore, in this study, we developed and validated a quantitative SYBR Green-based real-time RT-PCR assay for ARAV and RIOMV, being the most virulent among hantavirus genus and most commonly found in Brazil.

## 2. Materials and Methods

### 2.1. Serum Sample and Viral RNA Extraction

The RNA of samples of human and wild rodents was extracted using the QIAamp viral RNA extraction kit (Qiagen, Hilden, Germany) according to the manufacturer’s protocol. The RNA was recovered in 50 µL of RNase-free water with 40 units of recombinant RNase-OUT recombinant inhibitor (Invitrogen, Carlsbad, CA, USA) and stored at −70 °C.

### 2.2. Plasmid Cloning

An amplicon of ~264 nucleotides of the S segment of ARAV generated using primers, as described by Moreli *et al*. [16], was cloned in the pCR-TOPO 2.1 vector and introduced in *Escherichia coli* DH5α One Shot (Invitrogen, Carlsbad, CA, USA) according to the manufacturer’s protocol. From a selected transformant containing the desired construct, plasmid DNA was isolated using the QIAprep Spin Miniprep Kit (Qiagen, Hilden, Germany). The plasmid was sequenced to confirm the insertion and showed 99% sequence identity to the RNA S segment of ARAV (EF571895.1). 

### 2.3. *In Vitro* Transcription

Plasmids containing the insert were linearized by digestion with *Bam*H I enzyme (Fermentas, Vilnius, Lithuania). The linearized DNA plasmid was subsequently purified using the QIAquick PCR purification kit (Qiagen, Hilden, Germany) and *in vitro* transcribed using T7 RNA polymerase (Invitrogen, Carlsbad, CA, USA) following the manufacturer’s instructions. Residual DNA was removed by treatment with DNAse I (Invitrogen, Carlsbad, CA, USA) for 2 hours. Subsequently, RNA purification was performed using RNeasy kit (Qiagen, Hilden, Germany) and stored at −70 °C. In addition the products were subjected to PCR with primers described by Moreli *et al.* [16] to verify the complete degradation of plasmid DNA.

### 2.4. Standardization of the One-Step SYBR Green I Real-Time Quantitative RT-PCR

Quantitative RT-PCR was performed using the StepOnePlus™ Real-Time PCR System (Applied Biosystems, Foster City, CA, USA) and the SuperScript III Platinum One-Step qRT-PCR Kit (Invitrogen, Carlsbad, CA, USA) according to the manufacturer’s instructions. The optimal reaction mixture contained 2 µL of RNA template; 0.5 µL of MgSO_2_; 0.35 µL of each primer (ARAV forward and ARAV reverse [16]) (10 mM); 10 µL of SYBR buffer (2×); 0.5 µL of SuperScript III Platinum and 7.7 µL of MilliQ water in a final 20 µL volume. The temperature cycles to optimal reaction was 50 °C for 20 min for the production of cDNA; 95 °C for 5 min to activate the Taq polymerase and separate double-stranded DNAs; and 45 cycles at 95 °C for 15 s for denaturation; 55 °C for 25 s for primer annealing and 72 °C for 35 s for extension. The melting curve of the reaction (T_M_) was used to determine the specificity of amplified products. The T_M_ curve was obtained by performing a thermal cycle of 95 °C for 15 s, decreased to 60 °C for 1 min, and increased again to 95 °C for 15 s. The reactions were carried out in a 96-well optical reaction plates or MicroAmp reaction tubes (Applied Biosystems, Foster City, CA, USA). The size of all obtained amplicons was determined by electrophoresis in 1.5% agarose gel.

### 2.5. Quantitation of the ARAV RNA Based on a Standard Curve

RNA concentration was determined by spectrophotometry in a Thermo Scientific NanoDrop™ 1000 Spectrophotometer (NanoDrop Technologies, Wilmington, DE, USA). The measurements were performed in duplicate and the concentration, in copies/μL, was converted to copy number using the following formula: RNA copy number (copies/μL) = (RNA concentration (g/μL)/number of nucleotides of transcript × 340) × 6.022 × 10^23^. The linear range of quantitation of the one-step real-time RT-PCR assay was determined by using ten-fold serial dilutions of RNA transcribed by develop of standard curve. For each new reaction, a standard curve was developed based in dilutions of *in vitro*-transcribed RNA and generation of a standard curve. 

### 2.6. Specificity, Reproducibility and Detection Limit of the Reaction

The specificity of the one-step SYBR Green I quantitative RT-PCR was checked with other RNA viruses, including Coxsackie B5, Influenza A, Human Respiratory Syncytial virus A, Parainfluenza-2, Metapneumovirus, Oropouche and Rhinovirus-39. RIOMV in cell culture have also been tested. 

The reproducibility of the one-step SYBR Green real-time quantitative RT-PCR was analyzed based on the T_M_, slope and R_2_ values obtained from decimal dilutions (10^8^–10^3^ copies/μL) of the ARAV RNA *in vitro*-transcribed assayed in duplicate. Likewise, the detection limit of the reaction was determined by testing between 3.7 × 10^11 ^copies/μL to 3.7 × 10^−3 ^copies/μL of *in vitro* transcribed ARAV RNA in triplicate.

### 2.7. Comparison of the One-Step SYBR Green I Real-Time Quantitative RT-PCR with Conventional RT-PCR

The efficiency of amplification and detection of ARAV genome by the one-step SYBR Green real-time quantitative RT-PCR was compared with conventional RT-PCR [9]. We extracted the RNA from serum samples of HPS patients H1-H20 and macerated lung tissues from *Necromys lasiurus* rodents N1-N10 using the QIAamp viral RNA extraction kit (Qiagen, Hilden, Germany) and tested our method with both samples. In addition, the serum samples were tested for IgG and IgM to hantavirus via ELISA using the recombinant nucleoprotein (*N*) of ARAV as an antigen [17].

## 3. Results

### 3.1. The Standard Curve of the One-Step SYBR Green I Real-Time Quantitative RT-PCR

**Figure 1:**
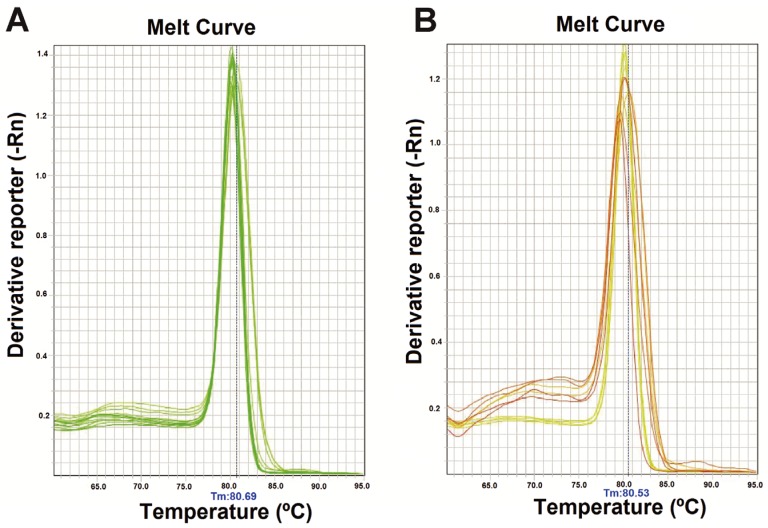
Melting peaks of the one-step SYBR Green I real-time RT-PCR. (**A**) Melting peaks of real-time RT-PCR obtained from *in vitro* transcribed ARAV RNA at 10^3^–10^8^ copies/µL; (**B**) Melting peaks obtained from all positive samples. All melting curves included in A and B have similar shapes.

The concentration of ARAV RNA *in vitro*-transcribed was 8.4 ng/μL, and it contained 3.7 × 10^11^ copies/μL of *in vitro* transcribed ARAV RNA. Equivalent ΔRn amplification plot curves were obtained when testing ARAV RNA at different dilutions in duplicate with values of 0.055305. Decimal dilutions of the ARAV RNA were tested, and their C_T_ values were plotted as the standard curve of the reaction. The parameters obtained for the SYBR Green I one-step real-time quantitative RT-PCR were: slope −3.075 (the ideal value is −3.200), percentage efficiency (EFF) 101.4%; correlation coefficient (R_2_) 0.995 (the ideal value is <1.0) and Y-inter 35.312. The C_T _values obtained from ARAV RNA dilutions were reproducible. Therefore, only one T_M_ peak between 80.83 ± 0.89 °C was observed for many of the tested ARAV RNA samples, as shown in the melting curves in Figure 1A and with the samples in Figure 1B.

### 3.2. Reproducibility, Detection Limit and Specificity of the Reaction

The reaction was reproducible based on triplicate analysis of *in vitro* transcribed ARAV RNA. Positive samples showed only a small variation in parameters: T_M_: 80.64 ± 0.67 °C; slope: 3.047 ± 0.068; R_2_: 0.977 ± 0.021 and C_T_ with variations of 3%–7% in each triplicate, as previously described by the manufacturers. The negative control was water or buffer instead of sample template (buffer EB). The positive control was ARAV RNA *in vitro*-transcribed, equivalent to that used in the standard curve. 

The detection limit of the test was 10 copies/µL of RNA based on triplicate analysis of decimal dilutions of *in vitro* transcribed ARAV RNA (3.7 × 10^11 ^copies/µL to 3.7 × 10^−3 ^copies/µL of *in vitro* transcribed ARAV RNA).

However, this one-step SYBR Green I real-time quantitative RT-PCR was able to amplify the genome of RIOMV, showing similar T_M_ peaks obtained for ARAV. The one-step SYBR Green I real-time quantitative RT-PCR did not amplify the genomes of others viruses including Coxsackie B5, Influenza A, Human Respiratory Syncytial virus A, Parainfluenza-2, Metapneumovirus, Oropouche and Rhinovirus-39.

### 3.3. Comparison of the One-Step SYBR Green Real-Time RT-PCR with Conventional RT-PCR

The results obtained by conventional RT-PCR were compared with those of the SYBR Green I one-step real-time quantitative RT-PCR, and real-time quantitative RT-PCR appeared to be a more sensitive method. A hantavirus genome was detected and quantified by real-time quantitative RT-PCR in two serum samples of HPS patients that were negative by conventional RT-PCR, as shown in Table 1. Five lung samples from wild rodents that were positive by conventional RT-PCR were also positive by the real-time quantitative RT-PCR. 

**Table 1:** Comparison of conventional RT-PCR and one-step SYBR Green I real-time RT-PCR for diagnosis of Araraquara virus.

N. S	IgG ELISA	IgM ELISA	RT-PCR	One-step SYBR Green I real-time RT-PCR Copies/µL of RNA ^1^	Conversion for Copies/mL of serum or Copies/mg of tissue
H1	IgG-12,800	Negative	Negative	Negative	Negative
H2	IgG-12,800	Negative	Negative	Negative	Negative
H3	IgG-1,600	Negative	Positive	6.93 × 10^3^ copies/µL	2.47 × 10^6^ copies/mL
H4	IgG-6,400	Negative	Negative	Negative	Negative
H5	IgG-3,200	Negative	Positive	8.85 × 10^2^ copies/µL	3.15 × 10^5^ copies/mL
H6	IgG-800	Negative	Positive	1.06 × 10^4^ copies/µL	3.78 × 10^6^ copies/mL
H7	IgG-3,200	Positive	Negative	1.03 × 10^2^ copies/µL	3.67 × 10^4^ copies/mL
H8	IgG-6,400	Positive	Negative	Negative	Negative
H9	IgG-1,600	Positive	Positive	2.72 × 10^3^ copies/µL	9.71 × 10^5^ copies/mL
H10	IgG-12,800	Positive	Negative	Negative	Negative
H11	IgG-6,400	Negative	Negative	7.4 × 10^1^ copies/µL	2.64 × 10^4^ copies/mL
H12	IgG-1,600	Negative	Positive	1.55 × 10^3^ copies/µL	5.53 × 10^5^ copies/mL
H13	IgG-1,600	Positive	Positive	1.34 × 10^3^ copies/µL	4.78 × 10^5^ copies/mL
H14	IgG-800	Positive	Positive	8.72 × 10^2^ copies/µL	3.11 × 10^5^ copies/mL
H15	IgG-6,400	Positive	Negative	Negative	Negative
H16	IgG-1,600	Positive	Positive	3.87 × 10^3^ copies/µL	1.38 × 10^6^ copies/mL
H17	IgG-3,200	Negative	Negative	Negative	Negative
H18	IgG-3,200	Negative	Negative	Negative	Negative
H19	IgG-400	Negative	Negative	Negative	Negative
H20	IgG-12,800	Negative	Negative	Negative	Negative
N1	-	-	Positive	1.39 × 10^3^ copies/µL	2.48 × 10^3^ copies/mg
N2	-	-	Negative	Negative	Negative
N3	-	-	Positive	8.9 × 10^4^ copies/µL	1.58 × 10^5^ copies/mg
N4	-	-	Positive	1.32 × 10^4^ copies/µL	2.35 × 10^4^ copies/mg
N5	-	-	Negative	Negative	Negative
N6	-	-	Negative	Negative	Negative
N7	-	-	Negative	Negative	Negative
N8	-	-	Positive	1.78 × 10^3^ copies/µL	3.17 × 10^3^ copies/mg
N9	-	-	Negative	Negative	Negative
N10	-	-	Positive	2.68 × 10^2^ copies/µL	4.78 × 10^2^ copies/mg

N. S: Number sample; H: Serum samples of HPS patients; N: Lung tissues from *Necromys lasiurus* rodents. ^1^ The hantavirus load of clinical samples obtained by plotting on a standard curve, expressed in copies/µL of RNA.

## 4. Discussion

PCR has been used extensively worldwide for the diagnosis of viral infections, including those caused by hantaviruses [9]. Real-time PCR has several advantages over conventional PCR. It is faster, more sensitive, and reproducible, allows for quantitative measurement, has lower hands-on-time and lower risk of contamination [9,18]. 

Recently, some quantitative real-time RT-PCRs, using TaqMan, were developed for quantitation of hantavirus [10,14,18]. Here, we report, for the first time, a quantitative real time SYBR Green-based technique for the detection and quantitation of ARAV and RIOMV, being an important cause of HPS in the Southeast and Central Plateau of Brazil [3]. 

The main reason for the choice of a detection method based on SYBR Green is that RNA viruses present a diagnostic challenge. After consecutive replications, RNA viruses can generate genomic alterations, making them difficult to detect. Thus, the use of TaqMan probes, which are very specific, may produce false negative diagnoses due to genomic alterations in the binding sites with the probe [19]. 

The one-step SYBR Green I real-time quantitative RT-PCR was highly sensitive, being able to detect 10 copies/µL of RNA. The test was also reproducible, based on equivalent melting curves and amplicon sizes obtained from hantavirus-infected samples in multiple reactions [13,20,21]. Moreover, the latter is a lower-cost reaction compared with other real-time RT-PCRs, such as TaqMan [9,21].

The amplicons obtained with the one-step SYBR Green I quantitative RT-PCR were undoubtedly part of the hantavirus genome because the primers used amplify part of the *N* gene of ARAV. This amplification occurs in a conserved region of the *N* gene; in fact, a previous analysis showed that these primers were also able to amplify the genomes of other South American hantaviruses [16]. Thus, the amplification of RNA from RIOMV showed T_M_ peaks similar to ARAV. 

The specificity of the reaction was confirmed by the T_M_ curve, which was consistently specific for the amplicon obtained; the mean peak Tm obtained with curves specific for hantavirus amplicons will enable easier discrimination of a primer-dimers and other viruses [13,20,21]. In addition, no amplification of genomes from other RNA viruses was obtained using the reaction. 

The standard curve of the SYBR Green I real-time quantitative RT-PCR was based on reactions using a vector containing 263 nucleotides of the S segment of the ARAV. The vector had been previously treated with DNAse I, preventing the amplification of remnants of plasmid DNA and erroneous measurements. Furthermore, DNAse I treatment prevented fluorophore binding to any sequence of double-stranded DNA [9,20]. The hantavirus load of clinical samples was quantified by plotting their C_T_ reaction values on a standard curve based on the C_T_ values of 10^8^–10^3^ copies/µL of ARAV RNA *in vitro*-transcribed [10,22].

The one-step SYBR Green I quantitative RT-PCR was more sensitive for hantavirus diagnosis than conventional RT-PCR. From the sera of 20 human HPS patients, a hantavirus genome was amplified by 10 by the one-step SYBR Green I quantitative RT-PCR, including two samples that were not amplified by conventional RT-PCR. These two samples had low viral loads (1.03 × 10^2^ and 7.4 × 10^1^ copies/µL of RNA or 3.67 × 10^4^ and 2.64 × 10^4^ copies/mL of serum) that were likely below the detection capacity of conventional RT-PCR (Table 1). 

The analysis of viral load obtained from serum samples of HPS patients, infected with ARAV, demonstrated high plasma levels of viral RNA during acute infection (2.64 × 10^4^–3.78 × 10^6^ copies/mL), similar to those found in others studies [23,24], which analyzed plasma viral load in patients infected with Sin Nombre virus and was found to range between 10^4.7^–10^7.5^ and 1.3 × 10^4^–1.8 × 10^6^ copies/mL (Table 1).

Our data corroborates with results that demonstrated high plasmatic viral load of Puumala virus and Dobrava virus in patients with hemorrhagic fever with renal syndrome (HFRS) and *Nephropathia epidemica* (NE) (10^2^–10^8^ and 1.71 × 10^2^–3 × 10^6^ copies/mL) [13,25]. These reports demonstrate that IgG antibody values are inversely correlated with virus concentration. This result can be an association between high antibody titles with low viremia (Table 1). 

The analysis of viral load obtained from macerated lung samples of *Necromys lasiurus* showed variable levels of viral RNA (4.78 × 10^2^–1.58 × 10^5^ copies/mg) (Table 1). A study with experimental infection of *Peromyscus maniculatus* showed that the highest titers of viral RNA are found 21 days post infection, and decline 60 days post infection [15].

The one-step SYBR Green I quantitative RT-PCR was performed more rapidly than conventional RT-PCR, taking approximately 2 h. This assay could be useful for studies of the pathogenesis of hantaviruses and for the analysis of the effect of antiviral drugs for ARAV and RIOMV [11,13]. Furthermore, it may be that this technique is able to detection of other hantavirus South American, due to the specificity of the primers used for these hantavirus described by Moreli *et al.* [16]. 
